# One-Step Fabrication of Water-Dispersible Calcium Phosphate Nanoparticles with Immobilized Lactoferrin for Intraoral Disinfection

**DOI:** 10.3390/ijms26020852

**Published:** 2025-01-20

**Authors:** Maki Nakamura, Ayako Oyane, Tomoya Inose, Yukimi Kanemoto, Hirofumi Miyaji

**Affiliations:** 1 Nanomaterials Research Institute, National Institute of Advanced Industrial Science and Technology (AIST), AIST Tsukuba Central 5, 1-1-1 Higashi, Tsukuba 305-8565, Japan; a-oyane@aist.go.jp (A.O.); t.inose@aist.go.jp (T.I.); 2 Periodontics, Hokkaido University Hospital, N14W5, Kita-ku, Sapporo 060-8648, Japan; yukimi-kanemoto@den.hokudai.ac.jp; 3 General Dentistry, Department of Oral Health Science, Faculty of Dental Medicine, Hokkaido University, N13W7, Kita-ku, Sapporo 060-8586, Japan; miyaji@den.hokudai.ac.jp

**Keywords:** calcium phosphate, lactoferrin, heparin, nanoparticles, antibacterial activity

## Abstract

Lactoferrin is a highly safe antibacterial protein found in the human body and in foods. Calcium phosphate (CaP) nanoparticles with immobilized lactoferrin could therefore be useful as intraoral disinfectants for the prevention and treatment of dental infections because CaP is a mineral component of human teeth. In this study, we fabricated CaP nanoparticles with co-immobilized lactoferrin and heparin using a simple one-step coprecipitation process. Heparin, a negatively charged polysaccharide, was used as both an immobilizing agent for lactoferrin and a particle-dispersing agent. The immobilization efficiency for lactoferrin in the CaP nanoparticles depended on the concentrations of both the lactoferrin and heparin in the reaction solution and was over 90% under optimal conditions. The nanoparticles had a hydrodynamic diameter of about 150–200 nm and could be well dispersed in water, owing to their relatively large negative zeta potential derived from heparin. They were found to exhibit antibacterial activity against *Actinomyces naeslundii*, which is involved in the initial formation of dental plaque that consequently leads to dental caries and periodontal disease. These results indicate the potential of the proposed nanoparticles as intraoral disinfectants.

## 1. Introduction

Lactoferrin is an iron-binding basic glycoprotein with an isoelectric point of 8.0–8.5 and a molecular mass of about 80 kDa that contains many basic amino acids, such as lysine and arginine. Lactoferrin is found in mammalian secretions such as milk, tears, and saliva and has multiple biological functions, including antibacterial, antiviral, antifungal, and antiparasitic activities [1,2,3]. Since lactoferrin is a highly safe antimicrobial protein widely used as an ingredient in foods, pharmaceuticals, and cosmetics [4], it has attracted attention in the dental field as an alternative to antibiotics for the prevention and treatment of dental caries and periodontal disease [5].

Oral bacteria that cause dental caries and periodontal disease often proliferate in tiny spaces in the oral cavity, such as dental fissures, gaps between the teeth, and periodontal pockets. Thus, nano- and micro-sized materials, which can immobilize a high content of lactoferrin, penetrate into these tiny spaces, and then release the lactoferrin, are expected to be useful as intraoral disinfectants. Calcium phosphate (CaP) nanoparticles are considered to be suitable for this purpose because they are biocompatible, biodegradable (for certain CaP phases), and chemically similar to the mineral components of teeth [6,7,8]. There have been previous reports on CaP nanoparticles with immobilized lactoferrin fabricated using a multi-step process involving the synthesis of CaP nanoparticles followed by lactoferrin adsorption [9,10,11,12]. However, such a multi-step process requires a long time. Furthermore, since these nanoparticles were produced for other purposes, their antibacterial activities against pathogenic oral bacteria were not investigated.

In the present study, rather than a multi-step process, we used a coprecipitation process involving a highly supersaturated CaP solution [7,8,13] in order to fabricate CaP nanoparticles with immobilized lactoferrin. Generally, coprecipitation processes can be completed in a single step and in a shorter time than adsorption-based multi-step processes. In addition, using the coprecipitation process, proteins can be immobilized both on the outer surface of CaP nanoparticles and in their interior, thus potentially enabling higher-capacity protein immobilization, compared with the case using superficial adsorption. Despite these advantages, a conventional coprecipitation process is less efficient at immobilizing basic proteins, such as lactoferrin, than acidic proteins, so a large amount of unused protein is left in the solution [14]. This is probably the reason why a multi-step adsorption-based process was used in previous studies [9,10,11,12].

We recently developed a coprecipitation process that achieved highly efficient immobilization of the basic proteins, cytochrome C and lysozyme, within CaP nanoparticles by co-immobilizing them with heparin, a negatively charged polysaccharide with numerous sulfo groups and a few carboxyl groups [15]. The presence of heparin in the nanoparticles also improved their water dispersibility, which is a requirement for intraoral disinfectants. We therefore hypothesized that heparin might play similar roles in the case of other basic proteins, such as lactoferrin.

In the present study, we investigated the fabrication of CaP nanoparticles with co-immobilized lactoferrin and heparin using one-step coprecipitation from highly supersaturated CaP solutions containing heparin and different concentrations of lactoferrin. The physicochemical properties of the resulting nanoparticles were evaluated, together with their lactoferrin immobilization efficiency. The results were compared with those for heparin-free nanoparticles with immobilized lactoferrin to elucidate the role of heparin. We also evaluated the potential of the nanoparticles fabricated by coprecipitation as intraoral disinfectants based on their antibacterial activity against oral bacteria. As an oral bacterium, we used *Actinomyces naeslundii* (*A. naeslundii*), which contributes to the initial formation of the dental plaque that leads to dental caries and periodontal disease [16].

## 2. Results

### 2.1. Morphological and Chemical Analyses of Products

**Figure 1:**
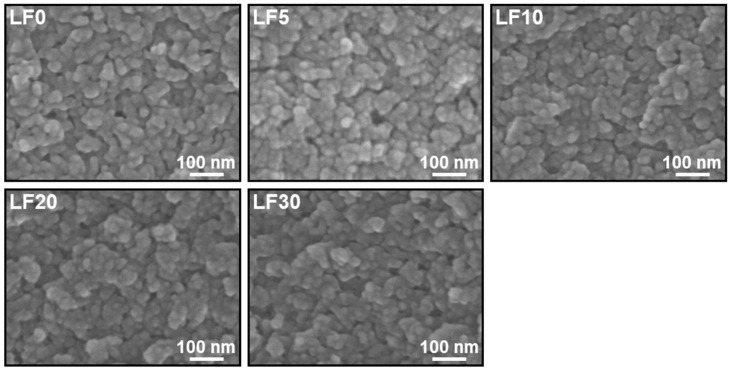
SEM images of **LF0**, **LF5**, **LF10**, **LF20**, and **LF30**.

**Figure 2:**
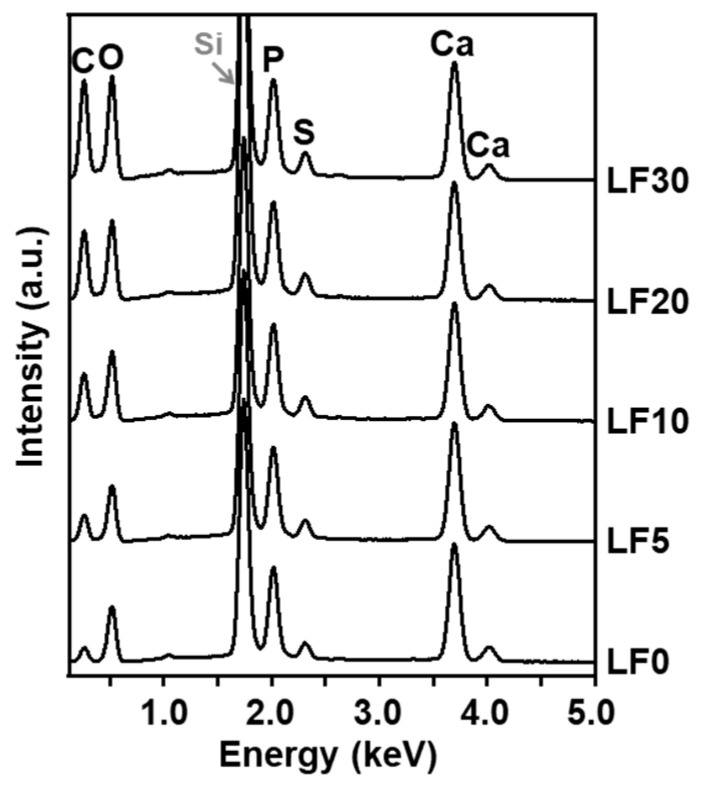
EDX spectra of **LF0**, **LF5**, **LF10**, **LF20**, and **LF30**. The Si peak was derived from the silicon substrate used for mounting the products.

**Figure 3:**
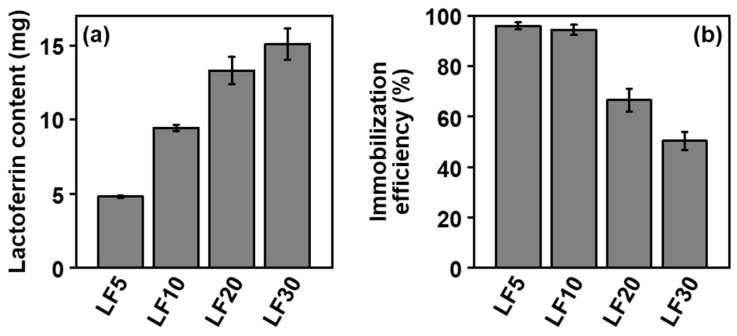
(**a**) Lactoferrin content and (**b**) lactoferrin immobilization efficiency for **LF5**, **LF10**, **LF20**, and **LF30** (average ± standard deviation, *n* = 3).

Various contents of lactoferrin were immobilized within CaP nanoparticles. First, we prepared five types of reaction solutions containing calcium chloride, dibasic potassium phosphate, sodium carbonate, heparin, and different concentrations of lactoferrin by mixing three solutions, A, B, and C (for details, see Section 4.1.). The products obtained after incubation for 24 h were referred to as **LF0**, **LF5**, **LF10**, **LF20**, and **LF30**, based on the concentration of lactoferrin in Solution C, 0, 5, 10, 20, and 30 mg/mL, respectively. The pH values (examined by a pH test paper) of the reaction solutions for preparing **LF0** and **LF10** were approximately 7.6 both before and after incubation. As shown in the scanning electron microscope (SEM) images (Figure 1), the products were irregularly shaped nanoparticles, regardless of the initial lactoferrin concentration. In the corresponding energy-dispersive X-ray spectroscopy (EDX) spectra (Figure 2), strong peaks associated with Ca and P were observed with similar intensities, indicating that all these nanoparticles were mainly composed of CaP. Peaks associated with C and O were also observed for all products, and their intensities relative to those of the Ca and P peaks increased with increasing lactoferrin concentration in the reaction solution (**LF0** < **LF5** < **LF10** < **LF20** < **LF30**). This was likely due to an increase in the lactoferrin content in the nanoparticles, consistent with the results of UV-visible spectroscopy (Figure 3a), which also showed such an increase. In addition to these EDX peaks, a small peak due to S, a component element of lactoferrin and heparin, was seen in the spectra of all products (Figure 2). The S peak in the spectrum of lactoferrin-free nanoparticles (**LF0**) was attributed to heparin, whereas those in the spectra of **LF5**, **LF10**, **LF20**, and **LF30** were attributed to both heparin and lactoferrin. As shown in Figure 3b, the immobilization efficiency for lactoferrin decreased with increasing lactoferrin concentration in the reaction solution. This trend was consistent with our previous report on CaP nanoparticles with immobilized another basic protein, cytochrome C [15]. In the preparation of **LF5** and **LF10**, over 90% of the lactoferrin in the reaction solution was immobilized within the nanoparticles, indicating that the loss of lactoferrin during preparation was less than 10%.

### 2.2. Nanoparticle Size Distribution and Zeta Potential

**Figure 4:**
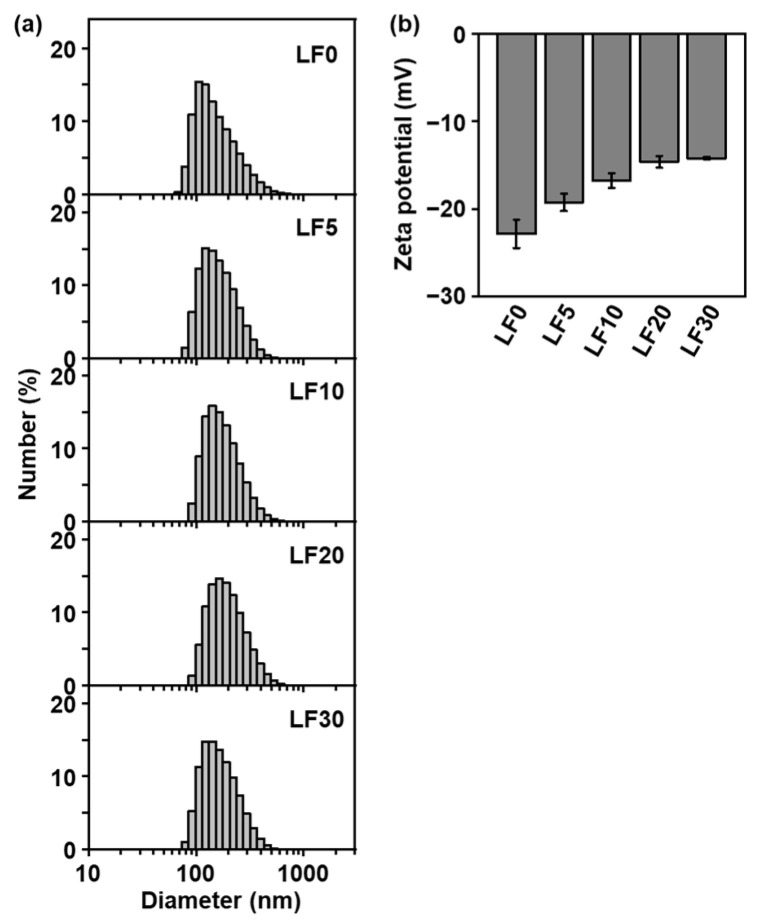
(**a**) Particle size distributions by number for **LF0**, **LF5**, **LF10**, **LF20**, and **LF30** obtained from DLS measurements and (**b**) corresponding zeta potentials obtained from ELS measurements (average ± standard deviation, *n* = 3). The nanoparticles were dispersed in water.

The CaP nanoparticles dispersed in water and had relatively large negative zeta potentials, irrespective of their lactoferrin content. Figure 4a shows particle size distributions for the different products dispersed in water, as determined by dynamic light scattering (DLS) measurements. All of the distributions were seen to be unimodal, with number-average hydrodynamic diameters of about 150–200 nm, indicating that the nanoparticles had similar sizes. The lactoferrin-free nanoparticles (**LF0**) exhibited the most strongly negative zeta potential of −22.9 mV (Figure 4b) by electrophoretic light scattering (ELS) measurement. This is thought to be due to the negatively charged heparin immobilized in the nanoparticles. It can be seen that the absolute value of the zeta potential decreased with increasing lactoferrin content. This is due to the partial neutralization of negatively charged heparin by positively charged lactoferrin in the nanoparticles and possibly also a reduction in the heparin content. Even for the product with the highest lactoferrin content (**LF30**), the zeta potential was still negative (−14.2 mV), sufficient for water dispersion. This suggests the presence of heparin on the nanoparticle surfaces. From **LF5** and **LF10**, which exhibited the highest immobilization efficiencies (Figure 3b), we selected **LF10** with the higher lactoferrin content (Figure 3a) for further investigation.

### 2.3. Microstructural and Crystalline Structural Analyses

**Figure 5:**
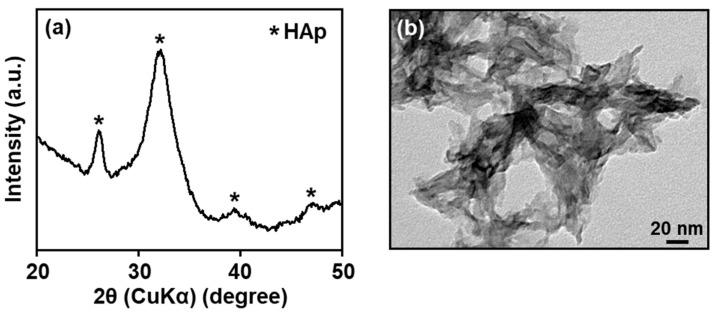
(**a**) XRD pattern and (**b**) TEM image of **LF10**.

The CaP in the nanoparticles (**LF10**) was low-crystalline hydroxyapatite (HAp). Figure 5a shows a thin-film X-ray diffraction (XRD) pattern for **LF10**, which exhibited peaks assigned to the HAp phase. The broadness of these peaks was an indication of the poor crystallinity of the HAp phase. The transmission electron microscope (TEM) image of **LF10** in Figure 5b showed the presence of irregularly shaped, angular nanocrystals with indistinct boundaries. This morphology was similar to that for HAp-based nanoparticles prepared by a similar precipitation process [15,17].

### 2.4. Comparison of Products with and Without Heparin

**Figure 6:**
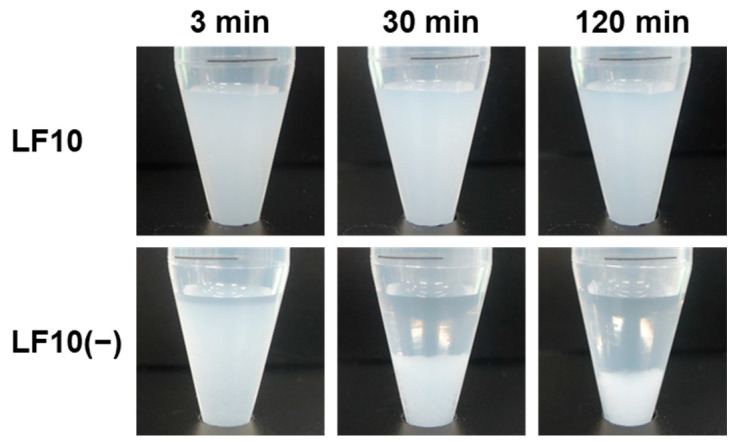
Digital photographs of **LF10** and **LF10(−)** dispersed in ultrapure water and allowed to stand for 3, 30, and 120 min.

The heparin in the nanoparticles acted as both an immobilizing agent for lactoferrin and a particle-dispersing agent, as reported for similar CaP nanoparticles with co-immobilized both heparin and cytochrome C or lysozyme, which are also basic proteins [15]. To elucidate these roles of heparin, a heparin-free product, referred to as **LF10(−)**, was prepared from a reaction solution containing lactoferrin (with the same lactoferrin concentration in Solution C as during the preparation of **LF10**) and no heparin, and various analyses were performed on **LF10** and **LF10(−)**; the results are shown in Table 1. According to a compositional analysis using inductively coupled plasma-optical emission spectroscopy (ICP-OES), it can be seen that there was little difference (<15%) between the Ca or P content in the two products obtained from a single batch of the reaction solution (4 mL). In contrast, the S content in **LF10** was approximately 7 times higher than that in **LF10(−)**, due to the presence of heparin in **LF10**. The small amount of S present in **LF10(−)** was due to the fact that lactoferrin also contains minor amounts of S in its chemical structure. The Ca/P molar ratio of **LF10** was close to that of stoichiometric HAp (1.67) and slightly higher than that of **LF10(−)**, suggesting the enhanced maturation of crystalline CaPs in **LF10**. The lactoferrin content and immobilization efficiency for **LF10** were seen to be approximately 1.5 times greater than those for **LF10(−)**. This indicates that the presence of heparin in the nanoparticles led to the enhanced immobilization of lactoferrin, most likely as a result of electrostatic attraction between the two. As shown in the digital photographs in Figure 6, when **LF10** and **LF10(−)** were dispersed in water, **LF10** maintained a dispersed state for at least 120 min, whereas **LF10(−)** started to settle within 3 min. This difference in dispersion stability reflects the different zeta potentials for the two products. As seen in Table 1, the zeta potential for **LF10** was strongly negative (−16.8 mV) because of the presence of heparin, but that for **LF10(−)** was slightly positive (2.4 mV). These results elucidate the role of heparin in **LF10** as a particle-dispersing agent.

### 2.5. Preliminary Antibacterial Assay

**Figure 7:**
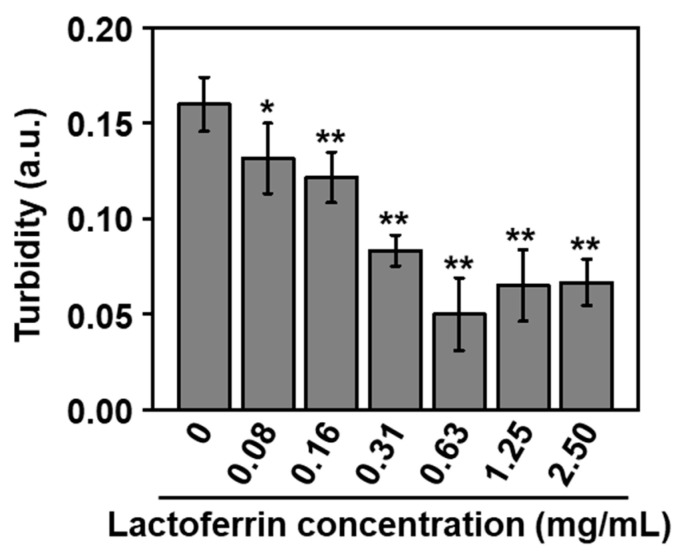
Turbidity of the bacterial (*A. naeslundii*) suspension after incubation with **LF10** at various concentrations of nominal lactoferrin for 24 h (average ± standard deviation, *n* = 6). * *p* < 0.05 and ** *p* < 0.01 relative to control (0 mg/mL).

The nanoparticles (**LF10**) exhibited antibacterial activity against *A. naeslundii* in a dose-dependent manner. Figure 7 shows the results of turbidity measurements of bacterial suspensions of *A. naeslundii* with different doses of **LF10**. The turbidity was seen to decrease with an increasing nominal lactoferrin concentration up to 0.63 mg/mL, after which it remained constant. Since turbidity is a measure of bacterial concentration, this indicates that the nanoparticles inhibited bacterial proliferation in a dose-dependent manner. It is presumed that lactoferrin was released from the nanoparticles via the dissolution of their mineral (HAp) component and exhibited antibacterial activity against *A. naeslundii*. It is known that *A. naeslundii* is an acid-producing bacterium [18], and the solubility of HAp increases in an acidic environment [19]. Hence, the proliferation of *A. naeslundii* may cause acidification of the bacterial suspension, thereby enhancing HAp dissolution and lactoferrin release from the nanoparticles. According to a previous report, the concentration of free lactoferrin that caused a 50% reduction in the viable count of *A. naeslundii* was about 5.7 mg/mL [20]. In the present study, the nanoparticles (**LF10**) exhibited antibacterial activity, even at the lower nominal lactoferrin concentration.

## 3. Discussion

### 3.1. Mechanism for Particle Formation

The formation mechanism for the CaP nanoparticles with co-immobilized lactoferrin and heparin was expected to be similar to that proposed in our previous report on CaP nanoparticles with co-immobilized cytochrome C and heparin [15]. First, homogeneous nucleation of CaP occurred instantly in the reaction solution, which was a highly supersaturated, nearly neutral CaP solution containing heparin and lactoferrin. Heparin carrying sulfo and carboxyl groups was immobilized within the thus-formed CaP nanoparticles through electrostatic interactions with calcium ions. The high affinity between heparin and CaP has been reported elsewhere [15,21]. Lactoferrin carrying positively charged basic amino acids, especially in its N1-domain [20,22], was also immobilized within the CaP nanoparticles. The presence of heparin in the reaction solution and/or nanoparticles enhanced lactoferrin immobilization due to electrostatic attraction at the positively charged heparin-binding sites of lactoferrin [22,23]. As in our previous report [15], the CaP nanoparticles just after coprecipitation (before incubation) were expected to be amorphous and spherical in shape. As seen in Figure 5, following 24 h of incubation, they grew into irregularly shaped, angular nanoparticles consisting of low-crystallinity HAp that immobilized both lactoferrin and heparin. As shown in Figure 3b, a lactoferrin immobilization efficiency of as high as 94% was achieved for **LF10**. Owing to the presence of heparin, the nanoparticles possessed a high charge density on their surfaces. This led to an electrostatic repulsion between the growing nanoparticles, which inhibited further aggregation. Consequently, size-regulated, water-dispersible CaP nanoparticles with co-immobilized lactoferrin and heparin were formed. Assuming that the HAp phase in **LF10** had the chemical formula Ca_10-*x*_(HPO_4_)*_x_*(PO_4_)_6-*x*_(OH)_2-*x*_ (*x* = 0.07, as estimated from the Ca and P contents in Table 1), the lactoferrin and heparin contents in **LF10** were estimated to be about 850 mg and 320 mg per 1 g of HAp, respectively. Under a similar assumption, the lactoferrin content in **LF10(−)** was estimated to be about 500 mg per 1 g of HAp, indicating again that heparin in **LF10** acted as an immobilizing agent for lactoferrin.

There have been several reports on the fabrication of CaP nanoparticles with immobilized lactoferrin using a multi-step process involving the synthesis of CaP nanoparticles followed by lactoferrin adsorption [9,10,11,12]. For example, Kim et al. prepared CaP nanoparticles modified with a heparin–dopamine conjugate, which subsequently adsorbed lactoferrin [11]. In contrast to such adsorption-based processes, the coprecipitation process used in the present study was a simple one-step process, in which lactoferrin was immobilized within CaP nanoparticles during their growth in the reaction solution. In addition to its simplicity, the present coprecipitation process offers advantages for producing CaP nanoparticles with a higher drug content. In fact, as mentioned above, the lactoferrin content in **LF10** was estimated to be about 850 mg per 1 g of HAp. This is remarkably high compared to the amount of lactoferrin that can be adsorbed onto HAp nanoparticles, which has been reported to be a maximum of 200 mg per 1 g of HAp [10]. The high lactoferrin content in **LF10** was made possible by the use of heparin as a lactoferrin-immobilizing agent (Table 1).

### 3.2. Potential Applications

The CaP nanoparticles with co-immobilized lactoferrin and heparin produced in the present study could be used as intraoral disinfectants. The nanoparticles consisted mainly of HAp, which is a mineral component of human teeth and a widely used biomaterial with a well-established safety record. The nanoparticles had a diameter of 150–200 nm and were dispersible in water (Figure 4a and Figure 6). Thus, they can penetrate deeply into tiny spaces, even at the micron scale, where oral bacteria are liable to proliferate and oral care cannot reach, such as dental fissures, gaps between the teeth, and periodontal pockets. They can then release lactoferrin via the partial dissolution of HAp and engage in antibacterial action against oral bacteria such as *A. naeslundii*, which is involved in the initial formation of dental plaque (Figure 7). Note that lactoferrin exhibits antibacterial activity against not only *A. naeslundii* but also other pathogenic oral bacteria that cause dental caries and periodontal disease [24,25,26,27]. Some oral bacteria have been known to acidify the surrounding environment and promote the decalcification of teeth. The nanoparticles are expected to have a secondary effect as an acid neutralizer, as reported for similar CaP-based particles [18,28]. Furthermore, the nanoparticles may have a tertiary effect as a remineralizing agent because they can also release calcium, phosphate, and hydroxide ions via the partial dissolution of HAp. Thus, these nanoparticles have potential as intraoral disinfectants for the prevention and treatment of dental caries and periodontal disease, although further investigation is needed.

## 4. Materials and Methods

### 4.1. Preparation of Nanoparticles

We prepared five types of reaction solutions containing calcium chloride, dibasic potassium phosphate, sodium carbonate, heparin, and different concentrations of lactoferrin. First, two source solutions were prepared: a 500 mmol/L sodium carbonate solution by dissolving sodium carbonate (FUJIFILM Wako Pure Chemical Corporation, Osaka, Japan) in ultrapure water and a 5 mg/mL heparin solution by dissolving heparin sodium (FUJIFILM Wako Pure Chemical Corporation) in a saline solution (OTSUKA NORMAL SALINE, Otsuka Pharmaceutical Co., Ltd., Tokyo, Japan). Three additional solutions (A, B, and C) were then prepared based on our previous report [15]. For Solution A, a 500 mmol/L calcium chloride solution (Calcium Chloride Corrective Injection 1 mEq/mL, Otsuka Pharmaceutical Co., Ltd.), a 5 mg/mL heparin solution, and a saline solution were mixed at a ratio of 8:25:17 (vol%). For Solution B, a 500 mmol/L dibasic potassium phosphate solution (Dibasic Potassium Phosphate Injection 20 mEq Kit, Terumo Corporation, Tokyo, Japan), a 500 mmol/L sodium carbonate solution, and a saline solution were mixed at a ratio of 4:4:17 (vol%). Solution C was prepared by dissolving lactoferrin (from bovine milk, FUJIFILM Wako Pure Chemical Corporation) in a saline solution at concentrations of 0, 5, 10, 20, and 30 mg/mL. The prepared solutions were held in a dry bath at a temperature setting of 25 °C. To prepare a reaction solution, 1 mL of Solution B, 1 mL of Solution C, and 2 mL of Solution A were added sequentially to a 15 mL centrifuge tube. The reaction solution (4 mL) was immediately mixed using a vortex mixer (Vortex-Genie 2, Scientific Industries, Inc., Bohemia, NY, USA) for 1 min and then incubated while shaking at 150 rpm in a thermostatic shaker (M·BR-104P, TAITEC CORPORATION, Koshigaya, Japan) at a temperature setting of 25 °C. After incubation for 24 h, the resulting product was collected via centrifugation (6000 rpm, 5 min) and washed three times with ultrapure water. Based on the concentration of lactoferrin in Solution C, the products were referred to as **LF0**, **LF5**, **LF10**, **LF20**, and **LF30**. At the same lactoferrin concentration of 10 mg/mL in Solution C, a product excluding heparin was also prepared in a similar manner without the addition of heparin; this was referred to as **LF10(−)**. The pH values of the reaction solutions for preparing **LF0** and **LF10** were examined by a pH test paper (Duotest pH 7.0–10.0, MACHEREY-NAGEL, Düren, Germany) before and after 24 h of incubation.

### 4.2. Characterization of Products

The products were mounted on a silicon substrate and dried under reduced pressure. The morphologies of **LF0**, **LF5**, **LF10**, **LF20**, and **LF30** were examined using SEM (S-4800, Hitachi High-Tech Corporation, Tokyo, Japan) after sputter coating with gold using a sputter coating machine (SC-701MkII, Sanyu Electron Co., Ltd., Tokyo, Japan). The chemical compositions of these products were determined by EDX (AZtecOne, Oxford Instruments plc, Abingdon, UK) in a tabletop SEM (TM4000Plus II, Hitachi High-Tech Corporation) system. The crystalline structure of **LF10** was investigated using XRD (MiniFlex600-C, Rigaku Corporation, Tokyo, Japan) with CuKα X-rays, and its nanostructure was examined by TEM (JEM-2100, JEOL Ltd., Tokyo, Japan) at an acceleration voltage of 200 kV. Prior to the TEM analysis, the product was mounted on a formvar-coated copper grid and dried under reduced pressure.

The lactoferrin content and immobilization efficiency for each product were determined as follows. After the first centrifugation following shaking for 24 h (see Section 4.1), the supernatant was removed and diluted with a saline solution. The amount of lactoferrin in the diluted supernatant solution was analyzed based on the optical absorbance at 279 nm, determined using UV-visible spectroscopy (UV-2450, SHIMADZU CORPORATION, Kyoto, Japan). The lactoferrin content in the product was calculated by subtracting the amount of residual lactoferrin in the supernatant from the total amount in the reaction solution. The lactoferrin immobilization efficiency for the product was calculated by dividing its lactoferrin content by the total amount of lactoferrin in the reaction solution. Three independent batches were used to obtain the average and standard deviation.

The particle size (hydrodynamic diameter) distribution and zeta potential for the products dispersed in ultrapure water were analyzed using DLS and ELS, respectively, with a particle size analyzer (Zetasizer Nano-ZS, Malvern Instruments Ltd., Worcestershire, UK). Before the DLS and ELS measurements, products obtained from a single batch of the reaction solution (4 mL) were dispersed in ultrapure water (10 mL) by sonication. The measurements were repeated three times per dispersion to obtain the average and standard deviation of the measured values. In parallel, 1 mL dispersions of **LF10** and **LF10(−)** in 15 mL centrifugation tubes were allowed to stand at room temperature for 3, 30, and 120 min. The dispersion state of the products was captured using a digital camera (COOLPIX P330, NIKON, Tokyo, Japan).

The Ca, P, and S contents in **LF10** and **LF10(−)** obtained from a single batch of the reaction solution (4 mL) were determined by chemical analysis as follows. The products were first dispersed in ultrapure water (10 mL) by sonication. To 0.5 mL of this dispersion, a 6 mol/L HCl solution (5 mL, FUJIFILM Wako Pure Chemical Corporation) and ultrapure water (4.5 mL) were added. After complete dissolution of the products and secondary dilution (only for Ca and P, 10 times) with 0.6 mol/L HCl solution, the Ca, P, and S contents in the HCl solutions were quantified using ICP-OES (ULTIMA2, HORIBA, Ltd., Kyoto, Japan). Similarly, the S content in 1 mg of lactoferrin and heparin was determined using ICP-OES. The S content derived from lactoferrin in **LF10** was estimated from the lactoferrin content and the S content in 1 mg of lactoferrin. The S content derived from heparin in **LF10** was calculated by subtracting the S content derived from lactoferrin from the total S content in **LF10**. Then, the heparin content in **LF10** was estimated from the S content in 1 mg of heparin and the S content derived from heparin in **LF10**. Three (**LF10**) or two (**LF10(−)**) independent batches were used to obtain the average and standard deviation.

### 4.3. Antibacterial Assay

The product **LF10** was added to a bacterial suspension of *A. naeslundii* (ATCC 27039, American Type Culture Collection, Manassas, VA, USA) (1.0 × 10^7^ CFU/mL) in amounts corresponding to lactoferrin concentrations of 0, 0.08, 0.16, 0.31, 0.63, 1.25, and 2.50 mg/mL. Brain heart infusion medium (Eiken Chemical Co., Ltd., Tokyo, Japan) was used as the culture medium. Each suspension was dispensed into the wells of a 48-well culture plate. After anaerobic incubation at 37 °C for 24 h, the turbidity of the culture medium was measured at a wavelength of 590 nm using a visible light spectrophotometer (CO7500 Colourwave, Funakoshi Co., Ltd., Tokyo, Japan) to detect bacterial growth.

### 4.4. Statistical Analysis

Statistical analysis was performed using data analysis and graphing software (OriginPro 2024, Lightstone Corp., Tokyo, Japan). The data were analyzed by a one-way analysis of variance followed by Tukey’s test. *p* < 0.05 was considered significant.

## 5. Conclusions

CaP nanoparticles with co-immobilized lactoferrin and heparin were fabricated by a simple one-step coprecipitation process. The presence of heparin in the nanoparticles enhanced the immobilization of lactoferrin, which led to an immobilization efficiency of over 90%. Heparin immobilized in the nanoparticles also improved their water dispersibility. The resulting nanoparticles had a hydrodynamic diameter of 150–200 nm that allows them to penetrate into tiny spaces in the oral cavity, such as dental fissures and periodontal pockets. The nanoparticles consisted mainly of HAp, which is a mineral component of human teeth, and exhibited antibacterial activity against *A. naeslundii*. From the above, the nanoparticles have potential as intraoral disinfectants for the prevention and treatment of dental caries and periodontal disease and warrant further study.

## Figures and Tables

**Table 1:** Ca, P, and S contents, Ca/P molar ratio, lactoferrin content, lactoferrin immobilization efficiency, heparin content, water dispersibility, and zeta potential for **LF10** and **LF10(−)** (average ± standard deviation, *n* = 3, except for *, where *n* = 2).

	LF10	LF10(−)
Ca content	110.9 ± 4.9 μmol	119.8 ± 1.2 μmol *
P content	67.0 ± 1.8 μmol	75.6 ± 0.0 μmol *
S content	15.3 ± 0.2 μmol	2.2 ± 0.1 μmol *
Ca/P ratio	1.66 ± 0.04	1.59 ± 0.02 *
Lactoferrin content	9.4 ± 0.2 mg	6.2 ± 0.2 mg
Lactoferrin immobilization efficiency	94 ± 2%	62 ± 2%
Heparin content	3.5 ± 0.1 mg	–
Water dispersibility (see Figure 6)	Good	Poor
Zeta potential	−16.8 ± 0.9 mV	2.4 ± 0.4 mV

## Data Availability

Data are contained within the article.
